# Withanolide-Type Steroids from *Physalis nicandroides* Inhibit HIV Transcription

**DOI:** 10.1021/acs.jnatprod.1c00637

**Published:** 2021-09-22

**Authors:** Vito A. Taddeo, Marvin J. Núñez, Manuela Beltrán, Ulises G. Castillo, Jenny Menjívar, Ignacio A. Jiménez, José Alcamí, Luis M. Bedoya, Isabel L. Bazzocchi

**Affiliations:** † Instituto Universitario de Bio-Orgánica Antonio González and Departamento de Química Orgánica, Universidad de La Laguna, Avenida Astrofísico Francisco Sánchez 2, 38206 La Laguna, Tenerife Spain; ‡ Dipartimento di Farmacia, Università degli Studi “G. d’Annunzio” Chieti-Pescara, Via dei Vestini 31, 66100 Chieti, Italy; § Laboratorio de Investigación en Productos Naturales, Facultad de Química y Farmacia, Universidad de El Salvador, Final Avenida de Mártires y Héroes del 30 de Julio, San Salvador 1101, El Salvador; ⊥ Retrovirus Laboratory, Department of AIDS Immunopathogenesis, National Centre of Microbiology, Instituto de Salud Carlos III, Ctra. Pozuelo Km. 2, 28220 Majadahonda, Madrid, Spain; ∥ Museo de Historia Natural de El Salvador, Ministerio de Cultura, San Salvador 1101, El Salvador; # Pharmacology, Pharmacognosy and Botany Department, Pharmacy Faculty, Universidad Complutense de Madrid, Pz. Ramón y Cajal s/n, 28040 Madrid, Spain

## Abstract

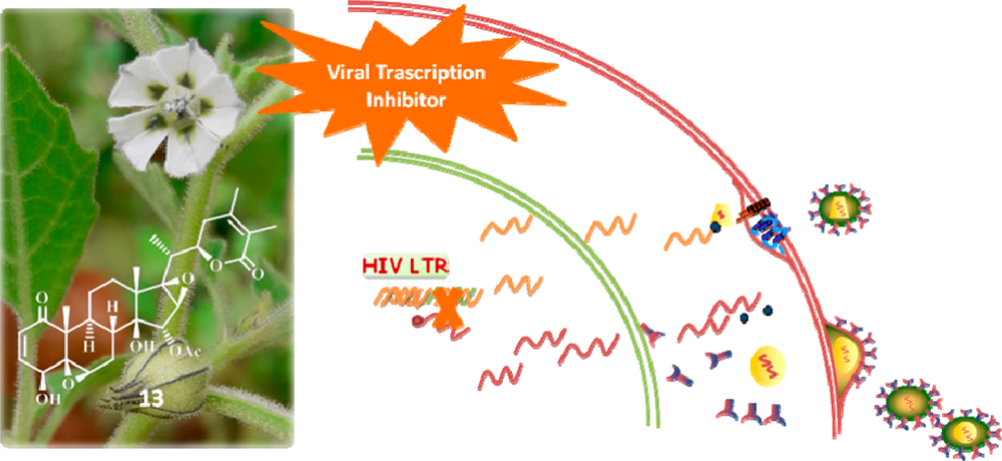

The aim of the present study is to report the isolation, structural elucidation, and antiviral evaluation of four new withanolide-type steroids, named nicansteroidins A–D (**1**–**4**), together with nine related known compounds (**5**–**13**) isolated from the aerial parts of *Physalis nicandroides*. Their structures were established based on an extensive spectroscopic analysis, including 1D and 2D NMR techniques. Outstandingly, nicansteroidins A and B possess an unusual side chain with an exocyclic double bond on the δ-lactone system, whereas nicansteroidins C and D have an uncommon cycloperoxide functionality in ring A as distinct structural motifs. Their biological evaluation as inhibitors of human immunodeficiency virus type 1 replication revealed that two compounds from this series, **7** and **13**, displayed strong inhibition of HIV-1 replication with IC_50_ values lower than 2 μM. Moreover, cellular mechanism experiments showed that the main target of these compounds in the HIV replication cycle is viral transcription. This study is the first report of withanolide-type steroids as HIV inhibitors and provides insight into their potential as candidates for further preclinical studies.

Acquired immunodeficiency syndrome/human immunodeficiency virus (AIDS/HIV) infection is still a pandemic around the world. According to the Joint United Nations Program on HIV/AIDS (UNAIDS), about 38 million people are living with HIV today, including 1.7 million people newly infected.^1^ Today, there is no vaccine, and although there are highly active antiretroviral therapies (ART), they have serious limitations such as complex treatment protocols, toxicity, and the emergence of viral resistance together with the persistence of latently infected cells not reached by ART, making lifelong treatment a necessity.^2^ Hence, the development of new antiretroviral drugs with novel structures and targets would be highly desirable. In this regard, natural products could be an efficient alternative to combat HIV infection, since they have demonstrated to be one promising option to discover and develop new antiretroviral drugs. In fact, the WHO has recommended that ethnomedicines and various other natural constituents be tested systematically to combat HIV.^3^

*Physalis* is a genus belonging to the Solanaceae family that comprises about 120 species, mainly distributed in American tropical and temperate regions. Most species from this genus have been used for a long time as traditional medicines in Asia and America to relieve a variety of illnesses, such as malaria, asthma, hepatitis, dermatitis, and liver disorders, and also have antimycobacterial, antitumor, antipyretic, and immunomodulatory effects.^4^ Various *Physalis* species are known in El Salvador as “turtle egg” or “miltomate” and have been used in the preparation of sauces and as diuretics.^5,6^ Phytochemical investigations have reported withanolides^7^ as the most frequently occurring constituents in *Physalis* species,^8^ which include physalins, C_28_ highly oxygenated C/D seco-steroids, that occur in *Physalis* and closely related genera belonging to the Solanaceae.^9^ In addition, labdane diterpenoids, flavonoids, sucrose esters, and ceramides^4^ have been reported from this genus. Previous phytochemical studies on *Physalis nicandroides* reported the characterization of sucrose esters,^10^ labdane diterpenoids,^11,12^ and withanolides.^12^

As a part of an intensive investigation toward the discovery of naturally occurring antiretroviral agents, the current research reports the isolation, structure characterization, and anti-HIV evaluation of 13 withasteroid-type metabolites from *P. nicandroides*. This study is the first report of withanolide-type steroids as potential agents against HIV infection.

## Results and Discussion

### Compound Structure Elucidation

**Scheme 1:**
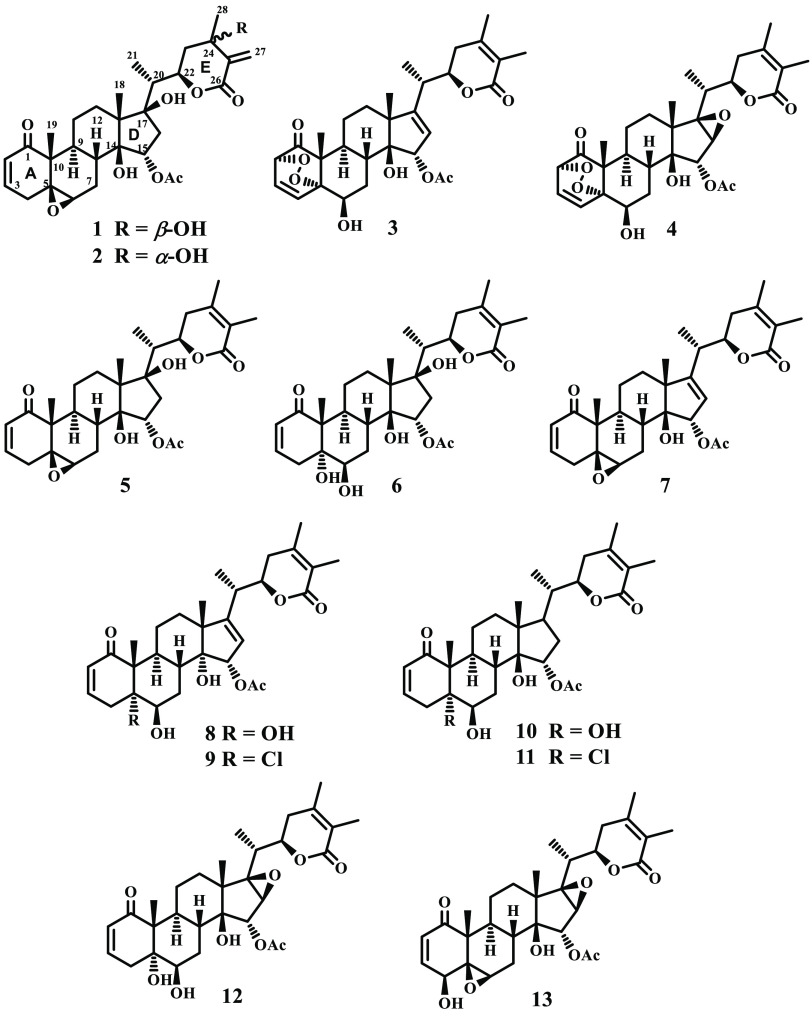
Structures of Withasteroids **1**–**13** Isolated from *Physalis nicandroides*

Repeated chromatography of the acetone extract of the aerial parts of *P. nicandroides* on silica gel and Sephadex LH-20, followed by preparative TLC, yielded four new withanolide-type steroids, named nicansteroidins A (**1**), B (**2**), C (**3**), and D (**4**), along with the known withanolides **5**–**13** (Scheme 1). Their structural elucidation was performed as described in the following paragraphs.

Compound **1** was isolated as a colorless lacquer and showed the molecular formula C_30_H_40_O_9_ by HREIMS, suggesting 11 degrees of unsaturation. The UV spectrum exhibited a strong absorption at 215 nm, indicating the presence of an α,β-unsaturated carbonyl system. Its IR absorption bands revealed the presence of hydroxy (3435 cm^–1^), α,β-unsaturated ester (1717 cm^–1^), α,β-unsaturated ketone (1672 cm^–1^), and epoxide (1247 cm^–1^) groups. The EIMS displayed peaks consistent with losses of water (*m*/*z* 526 [M^+^ – H_2_O]) and acetic acid (*m*/*z* 466 [M^+^ – H_2_O – CH_3_COOH]) and a base peak at *m*/*z* 124 (C_7_H_8_O_2_), characteristic of an α,β-unsaturated δ-lactone-withanolide, originated by the cleavage of the C-20/C-22 bond.^13^ Its ^1^H NMR spectrum (Table 1) displayed signals due to the presence of two tertiary methyls [δ_H_ 1.17 (s, H_3_-18) and 1.23 (s, H_3_-19)], one secondary methyl at δ_H_ 1.05 (d, *J* = 7.0 Hz, H_3_-21), and one methyl group on oxygenated carbon at δ_H_ 1.49 (s, H_3_-28). Three oxygenated methines [δ_H_ 3.25 (1H, d, *J* = 2.2 Hz, H-6), 4.65 (td, *J* = 2.5, 11.8 Hz, H-22), and 5.06 (dd, *J* = 3.4, 8.6 Hz, H-15)] and two deshielded geminal vinyl protons [δ_H_ 6.05 (s) and 6.64 (s), H_2_-27] were also observed in the ^1^H NMR spectrum. Moreover, signals at δ_H_ 6.02 (dd, *J* = 2.9, 10.0 Hz) and 6.87 (ddd, *J* = 2.3, 6.2, 10.0 Hz) could be attributed to the most common 2,3-enone system on ring A in withanolides.^14^ In addition, three singlet signals assigned to OH groups [δ_H_ 2.92 (HO-14), 3.45 (HO-17), and 2.93 (HO-24)] were observed. In accordance with the molecular formula, its ^13^C NMR spectrum displayed 28 carbon resonances, excluding those attributed to an acetyl unit, which were classified by an HSQC experiment (Table 2). Thus, carbon resonances were attributed to an 2,3-enone moiety [δ_C_ 203.4 (C-1), 128.8 (CH-2), and 144.9 (CH-3)] and an oxacyclopropane unit [δ_C_ 61.9 (C-5) and 63.6 (CH-6)], with a regiosubstitution pattern characteristic of the A/B-rings in withasteroids.^14^ Two oxygenated tertiary carbons [δ_C_ 86.7 (C-14), 86.0 (C-17)] and an oxygenated secondary carbon [δ_C_ 79.5 (CH-15)] were located on the D-ring. The carbon resonances at δ_C_ 76.8 (CH-22) and 81.3 (C-24) and those assigned to a δ-lactone carbonyl carbon [δ_C_ 166.3 (C-26)] and an exocyclic methylene group [δ_C_ 129.3 (CH_2_-27) and 139.0 (C-25)] occurred in the E-ring. The presence of an acetate group was confirmed by the 1D NMR spectra, showing signals at δ_H_ 1.99 (3H, s), and δ_C_ 21.4 (q) and 169.4 (s) (Tables 1 and 2). ^1^H–^1^H COSY and ^1^H–^13^C HSQC NMR experiments revealed four spin systems: CH-2/CH-3/CH_2_-4, CH-6/CH_2_-7, CH-15/CH_2_-16, and CH_3_-21/CH-20/CH-22/CH_2_-23. These data suggest that compound **1** is a withanolide-type steroid.

**Table 1:** ^1^H NMR Spectroscopic Data for Compounds **1**–**5**^a^

position	**1**	**2**	**3**	**4**	**5**
2	6.02, dd (2.9, 10.0)	6.02, dd (2.7, 10.1)	4.45, dd (1.1, 6.3)	7.00, dd (1.3, 8.4)	6.02, dd (2.6, 10.1)
3	6.87, ddd (2.3, 6.2, 10.0)	6.86, ddd (2.3, 6.1, 10.1)	6.62, dd (6.3, 8.4)	6.66, dd (6.5, 8.4)	6.86, ddd (2.6, 6.2, 10.1)
4	α 1.95, dd (6.2, 19.1) β 2.99, td (2.3, 19.1)	1.95, dd (6.1, 19.1) 3.00, td (2.3, 19.1)	7.02, dd (1.1,8.4)	4.48, dd (1.3, 6.5)	1.94, dd (6.2, 19.0) 2.98, td (2.6, 19.0)
6	3.25, d (2.2)	3.25, d (2.8)	4.03, s	4.08, s	3.24, d (2.8)
7	1.56,^b^ 2.37^b^	1.56,^b^ 2.36, td (3.1, 14.4)	1.80,^b^ 2.01^b^	1.58,^b^ 2.09^b^	1.54,^b^ 2.36, td (2.8, 14.5)
8	1.88, dt (3.3, 12.2)	1.87, dt (3.2, 12.0)	2.06^b^	1.94^b^	1.88, dt (3.3, 12.2)
9	2.16^b^	2.16, dt (4.4, 12.0)	2.61, dt (3.5, 12.4)	2.52, td (3.6, 12.4)	2.16^b^
11	1.46,^b^ 2.14^b^	1.54,^b^ 2.13^b^	1.39,^b^ 1.94^b^	1.38^b^	1.45,^b^ 2.14^b^
12	1.58^b^	1.55,^b^ 1.62^b^	1.50,^b^ 1.82^b^	1.59,^b^ 2.10^b^	1.53,^b^ 1.61^b^
15	5.06, dd (3.4, 8.6)	5.06, dd (3.5, 8.5)	5.30, d (2.5)	5.07, s	5.05, dd (3.5, 8.6)
16	1.81, dd (3.4, 16.0)	1.84, dd (3.5, 16.0)	5.63, d (2.5)	3.49, s	1.80, dd (3.5, 16.0)
	2.66, dd (8.6, 16.0)	2.65, dd (8.5, 16.0)			2.65, dd (8.6, 16.0)
18	1.17, s	1.20, s	1.16, s	1.15, s	1.12, s
19	1.23, s	1.25, s	1.18, s	1.20, s	1.24, s
20	2.27, dq (4.6, 7.0)	2.25, dq (4.6, 7.1)	2.50, q (7.0)	2.60, dq (4.8, 7.2)	2.24, dq (4.5, 7.0)
21	1.05, d (7.0)	1.00, d (7.1)	1.10, d (7.0)	1.01, d (7.2)	1.06, d (7.0)
22	4.65, td (2.5, 11.8)	5.01, td (2.6, 12.1)	4.30, ddd (3.6, 7.4,11.3)	4.49, ddd (3.5, 4.8, 12.8)	4.70, td (3.5, 12.8)
23	2.34^b^	2.62, dd (1.7, 15.4)	2.29, d (16.6)	2.12, d (3.5, 15.0)	2.42, dd (2.4, 16.6)
		1.75, dd (11.8, 15.4)	2.40, t (16.6)	2.39, t (15.0)	2.51, t (16.6)
27	6.05, s; 6.64, s	6.08, s; 6.72, s	1.88, s	1.88, t (1.1)	1.87, s
28	1.49, s	1.55, s	1.98, s	1.94^b^	1.92, s
OAc-15	1.99, s	1.99, s	2.11, s	2.21^b^	1.98, s
OH-14	2.92, s	2.55, br s		3.21, br s	2.79, s
OH-17	3.45, s	3.57, s			3.30, s
OH-24	2.93, s	8.06, s			

a Spectra recorded in CDCl_3_ at 600 MHz (*J* are given in parentheses in Hz). Data based on COSY, HSQC, and HMBC experiments.

b Signals without multiplicity assignments were overlapping resonances deduced by HSQC experiments.

**Table 2:** ^13^C NMR Spectroscopic Data for Compounds **1**–**5**^a^

position	**1**	**2**	**3**	**4**	**5**
1	203.4, C	203.1, C	206.8, C	206.2, C	203.4, C
2	128.8, CH	128.8, CH	78.6, CH	78.8, CH	129.2, CH
3	144.9, CH	144.8, CH	126.3, CH	126.0, CH	145.1, CH
4	32.9, CH_2_	32.9, CH_2_	142.2, CH	141.9, CH	33.3, CH_2_
5	61.9, C	62.0, C	84.1, C	83.9, C	83.9, C
6	63.6, CH	63.7, CH	67.0, CH	67.3, CH	62.2, CH
7	24.5, CH_2_	24.5, CH_2_	27.9, CH_2_	29.8, CH_2_	24.8, CH_2_
8	35.1, CH	35.3, CH	34.6, CH	34.4, CH	35.5, CH
9	38.2, CH	38.3, CH	38.2, CH	37.4, CH	38.6, CH
10	48.3, C^b^	48.3, C	48.5, C	48.1, C	48.7, C
11	22.6, CH_2_	22.6, CH_2_	21.9, CH_2_	20.7, CH_2_	22.8, CH_2_
12	30.4, CH_2_	30.2, CH_2_	38.5, CH_2_	32.4, CH_2_	30.3, CH_2_
13	50.4, C	50.7, C	52.3, C	46.8, C	50.8, C
14	86.7, C	86.8, C	82.2, C	81.6, C	87.1, C
15	79.5, CH	79.5, CH	83.1, CH	76.8, CH^b^	79.9, CH
16	48.3, CH_2_^b^	47.9, CH_2_	120.6, CH	59.3, CH	48.6, CH_2_
17	86.0, C	86.1, C	161.5, C	76.3, C	86.4, C
18	15.5, CH_3_	14.5, CH_3_	16.7, CH_3_	15.9, CH_3_	15.1, CH_3_
19	14.9, CH_3_	15.0, CH_3_	19.0, CH_3_	18.5, CH_3_	15.2, CH_3_
20	41.9, CH	41.1, CH	35.6, CH	33.5, CH	42.2, CH
21	9.0, CH_3_	9.2, CH_3_	17.6, CH_3_	13.5, CH_3_	10.0, CH_3_
22	76.8, CH	75.2, CH	78.9, CH	76.8, CH^b^	77.1, CH
23	33.7, CH_2_	34.3, CH_2_	32.8, CH_2_	32.5, CH_2_	32.3, CH_2_
24	81.3, C	80.2, C	150.3, C	149.3, C	150.7, C
25	139.0, C	136.7, C	121.8, C	122.1, C	121.8, C
26	166.3, C	165.7, C	167.7, C	166.4, C	167.4, C
27	129.3, CH_2_	132.3, CH_2_	12.5, CH_3_	12.6, CH_2_	12.7, CH_3_
28	25.1, CH_3_	23.4, CH_3_	20.8, CH_3_	20.6, CH_3_	20.9, CH_3_
OAc-15	169.4, C	169.4, C	170.6, C	170.2, C	169.7, C
	21.4, CH_3_	21.4, CH_3_	21.3, CH_3_	22.8, CH_3_	21.7, CH_3_

a Spectra recorded in CDCl_3_ at 150 MHz. Data based on DEPTs, HSQC, and HMBC experiments.

b Overlapping signals.

Comparison of the NMR data of **1** with those obtained for physagulide I (**5**),^15^ the major component isolated in the present study (Tables 1 and 2), revealed that the substituent pattern of the cyclopentano-perhydrophenanthrene system is common to **1** and **5**. The structural differences between those compounds were a deshielded exocyclic methylene group [δ_H_ 6.05 (s) and 6.64 (s); δ_C_ 129.3 and 139.0], a methyl group attached to an oxygen-bearing carbon [δ_H_ 1.49 (s); δ_C_ 81.3 (C-24), 25.1 (CH_3_-28)], and an ester carbon at δ_C_ 166.3 in **1** vs a δ-lactone group in compound **5**, suggesting that **1** is the 24-hydroxy-25(27)-ene derivative of **5**.

**Figure 1:**
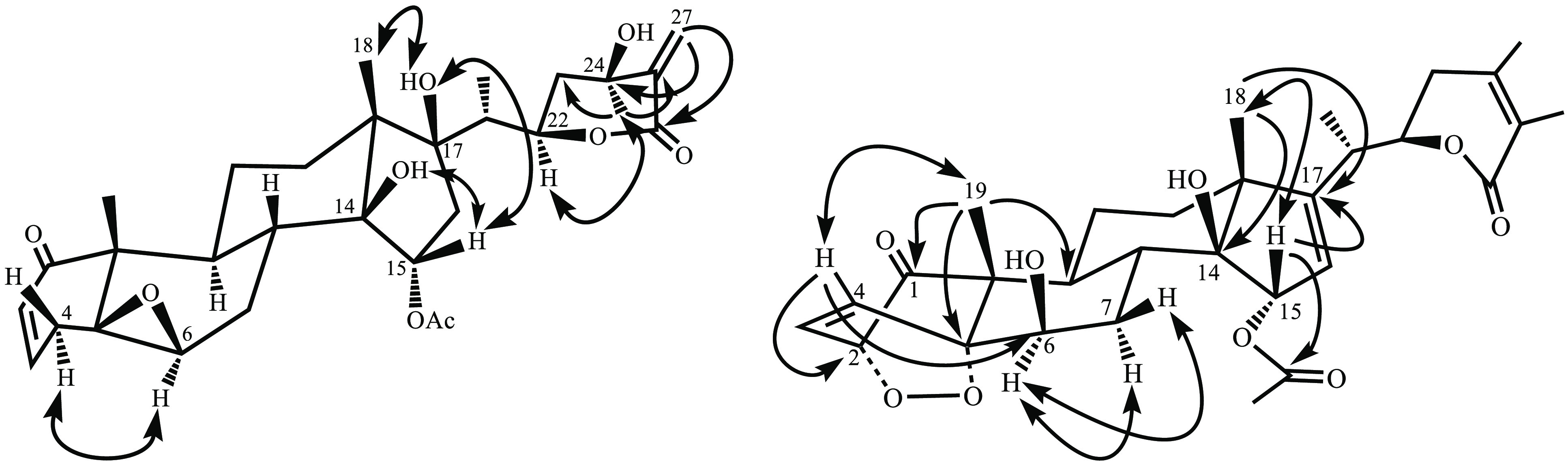
Selected HMBC (^1^H–^13^C) long-range correlations (→) and NOE effects (↔) for compounds **1** (left) and **3** (right).

The above-mentioned structural features were confirmed by the 2D NMR spectra. Thus, the regiosubstitution of **1** was confirmed by an HMBC experiment (Figure 1). The most relevant long-bond correlations were those of the signals at δ_H_ 6.05 and 6.64 (CH_2_-27) with the resonances at δ_C_ 166.3 (C-26), 139.0 (C-25), and 81.3 (C-24) and correlations of the signal at δ_H_ 1.49 (CH_3_-28) with the resonances at δ_C_ 33.7 (C-23), 81.3 (C-24), and 139.0 (C-25). These long-range correlations were used to locate the α,β-unsaturated δ-lactone, exocyclic methylene group, and tertiary hydroxy group in ring E. The relative configuration of **1** was established on the basis of the coupling constants and molecular mechanics calculation using the PC model^16^ and confirmed by a ROESY experiment. Thus, the β relative stereochemistry of the C-5–C-6 epoxide was confirmed by the cross-peak between H-4α and H-6α in a ROESY experiment (Figure 1). Correlations from a signal assigned to HO-14 with H-15/OH-17/H_3_-18 confirmed the α-stereochemistry of the acetate group at C-15 and the β-stereochemistry of the hydroxy groups at C-14 and C-17, whereas correlation from the Me-28 to H-22 indicated a β-orientation of the tertiary alcohol at C-24. Thus, the structure of **1** was established as 15α-acetoxy-5β,6β-epoxy-14β,17β,24β-trihydroxy-1-oxo-witha-2,25(27)-dien-26,22-olide, which has been named nicansteroidin A.

Compound **2** was assigned an identical molecular formula (C_30_H_40_O_9_) to that of **1** by HREIMS. Comparison of their NMR data showed as the most significant differences the downfield shift of signals assigned to H-22 (Δδ +0.36) and H-23 (Δδ +0.28) in the ^1^H NMR spectrum and upfield shifts of C-22 (Δδ −1.6), C-24 (Δδ −1.1), C-25 (Δδ −2.3), and C-28 (Δδ −1.7) and, in particular, the downfield shift of C-27 (Δδ +3.0) in the ^13^C NMR spectrum of compound **2**. These data suggested that the structural differences between these compounds involve the E-ring. 2D NMR (COSY, ROESY, HSQC, and HMBC) experiments allowed the complete and unambiguous assignment of chemical shifts, regiosubstitution pattern, and relative configuration, indicating that **2** is an epimer of **1** at C-24 on the side chain. This was confirmed by a ROESY experiment, showing NOE effects between OH-24 (δ_H_ 2.93) and H-20 (δ_H_ 2.25). Accordingly, the structure of **2** (nicansteroidin B) was established as 15α-acetoxy-5β,6β-epoxy-14β,17β,24α-trihydroxy-1-oxo-witha-2,25(27)-dien-26,22-olide.

These two compounds have one feature of particular interest since, to the best of our knowledge, they represent the first examples of withanolides with a 24-hydroxy-25(27)-en-α,β-unsaturated δ-lactone system on the side chain. The withametelins and withajardins^7^ are closely related to **1** and **2**, since they contain common structural motifs. However, in both these additional withanolide types the C-21 is directly bonded to C-25 or C-24, giving rise to a bicyclic lactone side chain with a six-membered heterocycle or homocycle, respectively.^7^

Nicansteroidin C (**3**) was isolated as a colorless lacquer, and the molecular formula was established as C_30_H_38_O_9_ on the basis of the sodiated molecular [M + Na] ion peak at *m*/*z* 565.2415 in its HRESIMS, suggesting an index of hydrogen deficiency of 12. The UV spectrum exhibited a strong absorption at 213 nm, indicating the presence of an α,β-unsaturated carbonyl system, whereas the IR absorption bands suggested the presence of hydroxy (3480 cm^–1^), carbonyl (1734 cm^–1^), and epoxide (1224 cm^–1^) groups. Its ^1^H NMR spectrum (Table 1) displayed signals for five methyl singlets at δ_H_ 1.16, 1.18, 1.88, 1.98, and 2.11 and a methyl doublet at δ_H_ 1.10 (d, *J* = 7.0 Hz) as the most characteristic downfield signals. In addition, four oxymethine protons [δ_H_ 4.03 (s), 4.30 (ddd, *J* = 3.6, 7.4, 11.3 Hz), 5.30 (d, *J* = 2.5 Hz) and 7.02 (dd, *J* = 1.1, 8.4 Hz)] and three vinyl protons [δ_H_ 5.63 (d, *J* = 2.5 Hz), 6.62 (dd, *J* = 6.3, 8.4 Hz), and 7.02 (dd, *J* = 1.1, 8.4 Hz)] were observed as the most upfield signals. In accordance with the molecular formula, 30 carbon resonances were resolved in the ^13^C NMR spectrum (Table 2) and characterized by an HSQC experiment as six methyls, four methylenes, 10 methines, and 10 quaternary carbons, including one keto carbonyl (δ_C_ 206.8), two ester carbonyls (δ_C_ 170.6 and 167.7), two oxygen-bearing carbons (δ_C_ 84.1 and 82.2), and three vinyl carbons (δ_C_ 121.8, 150.3, and 161.5). The aforementioned data accounted for 11 out of the 12 degrees of unsaturation, suggesting that compound **3** has an additional ring on a tetracyclic withanolide-type steroid skeleton, and the presence of a six-membered cyclic peroxide was the only option that satisfies the chemical shift requirements in the NMR spectra. A ^1^H–^1^H COSY experiment allowed the identification of six spin-coupling systems in the steroidal skeleton: CH-2/CH-3/CH-4 in ring A, CH-6/CH_2_-7 in ring B, CH-15/CH-16 in ring D, and CH-20/CH_3_-21, CH-20/CH-22, and CH-22/CH_2_-23 for the side chain. Moreover, the structure elucidation of **3** was also helped greatly by comparison of its spectroscopic data with those of previously reported witha-endoperoxides,^17^ confirming the presence of a six-membered cyclic peroxide ring system at C-2/C-5. The regiosubstitution of **3** was confirmed by the *J*_2,3_ long-range correlations observed in an HMBC experiment (Figure 1), showing as the most relevant correlations those between CH_3_-19 (δ_H_ 1.18) and the signals at δ_C_ 209.8 (C-1), 84.1 (C-5), 38.2 (C-9), and 48.5 (C-10) and those of H-4 (δ_H_ 7.02) with signals at δ_C_ 78.6 (C-2), 84.1 (C-5), 67.0 (C-6), and 48.5 (C-10), hence establishing the regiosubstitution on rings A/B. Moreover, three-bond correlations from the H_3_-18 (δ_H_ 1.16) to carbon resonances at δ_C_ 161.5 (C-17) 82.2 (C-14), and 38.5 (C-12) and those between H-15 (δ_H_ 5.30) and the carbon resonances at δ_C_ 52.3 (C-13) and 161.5 (C-17) and the carboxyl carbon at δ_C_ 170.6 confirmed the functional group substitution of ring D. The relative configuration of **3** was established on the basis of the coupling constants observed and confirmed by a ROESY experiment (Figure 1). Thus, the relative stereochemistry of the C-2/C-5 cyclic peroxide moiety was deduced by a correlation of H_3_-19 with H-4, which is indicative of the 2α,5α configuration of the endoperoxide moiety. Moreover, a cross-peak of H-6α with H-7α and H-7β confirmed the β-axial orientation of the hydroxy group at C-6, whereas correlations of H-15 with H_3_-18 defined the β-stereochemistry of the acetate group on ring D. All of these data and comparison with reported data^17,18^ established the structure of **3** (nicansteroidin C) as 15α-acetoxy-6β,14β-dihydroxy-1-oxo-2α,5α-dioxy-witha-3,16,24-trien-26,22-olide.

Nicansteroidin D (**4**) gave a molecular formula of C_30_H_40_O_10_ by HREIMS, indicating the presence of 11 degrees of unsaturation and one more oxygen atom than compound **3**. Their ^1^H and ^13^C NMR data (Tables 1 and 2) were strikingly similar. Thus, the most notable differences were the presence of signals for an oxacyclopropane group at C-16/C-17 [δ_H_ 3.49 (s) and δ_C_ 59.3 (CH), 76.8 (C)] in compound **4**, instead of the signals corresponding to a double bond in **3** [δ_H_ 5.63 (d, *J* = 2.5 Hz) and δ_C_ 120.6 (CH), 161.5 (C)]. 2D NMR experiments allowed the complete and unambiguous assignments of the chemical shifts, regiosubstitution, and relative configuration of compound **4**. Thus, the HMBC experiment determined the C-16/C-17 regiosubstitution of the oxacyclopropane moiety by the observed *J*_2,3_ long-range correlations of H-16 with C-17 (δ_C_ 76.3), C-20 (δ_C_ 33.5), C-10 (δ_C_ 48.1), C-15 (δ_C_ 76.8), and C-5 (δ_C_ 83.9), whereas a NOE effect from H-16 to H_3_-21 in a ROESY experiment defined its β-steroechemistry. Moreover, NOE interactions of H-2 to H_3_-19, H-6 to H-7α/H-7β, and that of H-15 to H_3_-18 defined the stereochemistry of the endoperoxide, hydroxyl group, and acetate moieties. This spectroscopic evidence supported the structure of compound **4** (nicansteroidin D) as 15α-acetoxy-16β,17β-epoxy-6β,14β-dihydroxy-1-oxo-2α,5α-dioxy-witha-3,24-dien-26,22-olide.

Cycloendoperoxide withanolides are very sporadically occurring natural products,^19^ and to date only nine highly oxygenated withanolides with an endoperoxide bridge have been characterized from plant species,^17,20−23^ among which physalin K,^20^ physalin Q,^20^ and physalinol A^21^ have been reported from the genus *Physalis*, particularly *Physalis alkekengi* var. *francheti*, which may have some biogenetic implications. These natural cycloendoperoxides have been explained via a [4 + 2] Diels–Alder cycloaddition^24^ between a cyclohexa-2,4-dien-1-one substrate and *in situ*-formed singlet oxygen, mainly produced by light absorption of photosensitizers in the plants.^25^ Although significant efforts have been made to identify endoperoxide biosynthetic enzymes, there is not enough evidence to ensure that cycloendoperoxides could be formed via an enzymatic process.^26^

The absolute configurations of compounds **1**–**4** were proposed by a biogenetic approach, based on them each having the same steroid core and specific rotation sign as the occurring known withanolide, also isolated in the present study, physagulin C (**12**) ([α]^25^_D_ +51.3, *c* 0.42, MeOH), for which the absolute configuration was determined by X-ray crystallographic analysis.^27^

The structures of the known compounds were identified by spectroscopic methods and comparison with reported data as follows: physagulin K (**6**),^28^ physagulin A (**7**),^29^ withaminimin (**8**),^18,30^ physagulin B (**9**),^29^ physagulin J (**10**),^30^ (20*S*,22*R*)-15α-acetoxy-5α-chloro-6β,14β-dihydroxy-1-oxowitha-2,24-dienolide (**11**),^31^ physagulin C (**12**),^27^ and physagulin F (**13**).^32^ The detailed ^1^H and ^13^C NMR (Tables 1 and 2) assignments of the one known compound, physagulide I (**5**),^33^ which have not been reported previously, were made using 1D and 2D NMR techniques (Tables 1 and 2).

### Biological Evaluation

Despite a great deal of research conducted on the therapeutic potential of withanolides,^7^ and in particular those isolated from *Physalis* species,^6^ to the best of our knowledge there is only one report available on steroidal anti-HIV pharmacophores in plant-based natural products.^3^ A lack of prior research studies on the antiviral properties of this pharmacologically active type of plant metabolites, and an ongoing search for new candidates for HIV infection treatment, encouraged us to evaluate the isolated withanolides for their inhibitory effect on HIV-1 replication. Compounds **6** and **11** could not be assessed due to their small amounts obtained, which precluded full biological evaluation.

**Figure 2:**
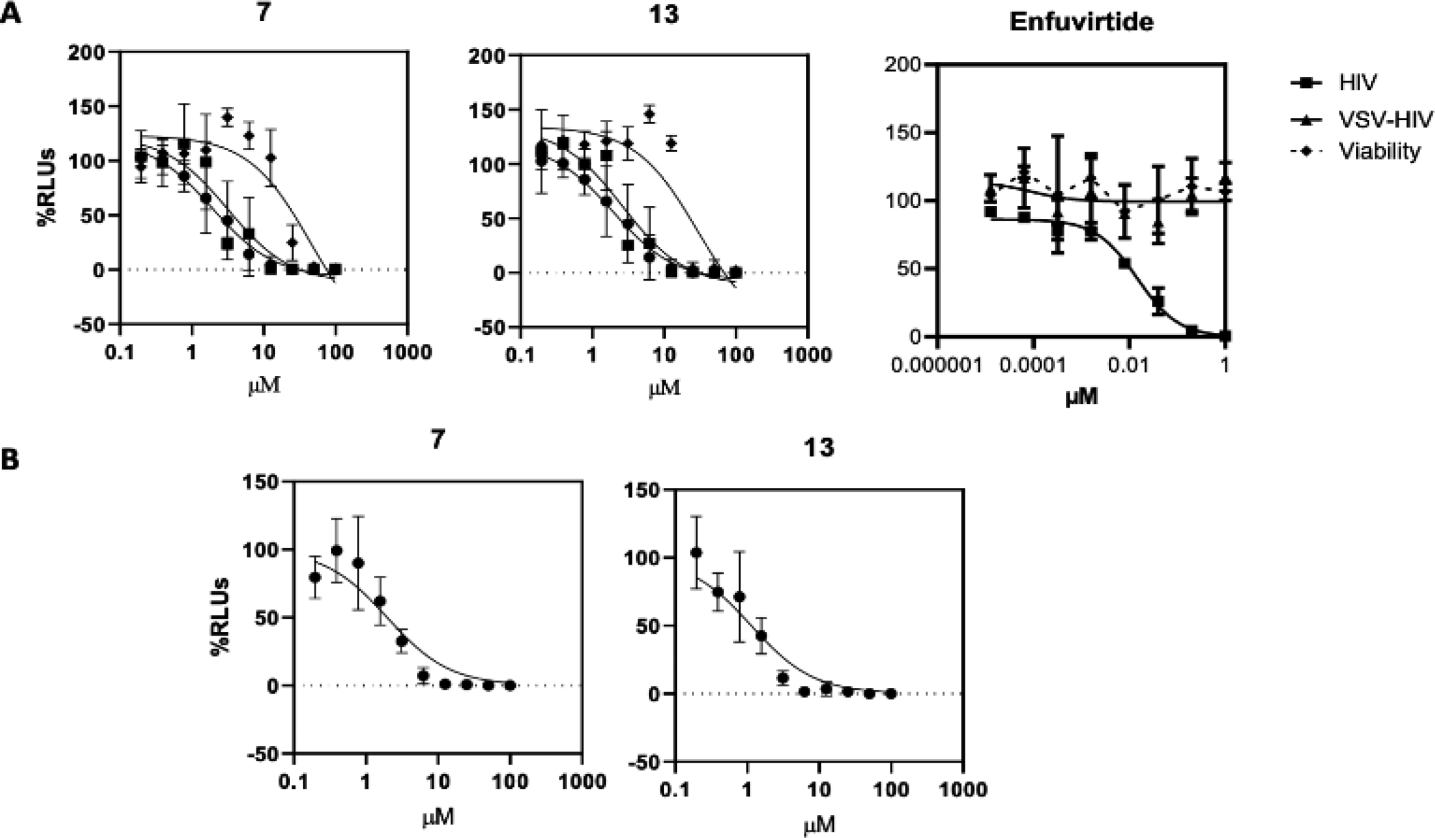
(A) In vitro evaluation of the anti-HIV activity and cytotoxicity of isolated compounds. MT-2 cells were infected with an X4 recombinant HIV (NL4.3-Ren, 100.000 RLUs or 20 ng p24/well) or with a VSV pseudotyped HIV (VSV-HIV, 100.000 RLUs or 20 ng p24/well) in the presence of different concentrations of test compounds or the fusion inhibitor enfuvirtide for 48 h. The same concentration of vehicle (DMSO or water) was used as a nontreated control (100%). The cell culture was then lysed, and relative luminescence units (RLUs) were measured in a luminometer. Cell viability was evaluated in mock-infected cells in parallel using the CellTiter Glo reagent (Promega). (B) Effect of isolated compounds on HIV transcription. MT-2 cells were transfected with a pNL4.3-Luc plasmid (1 ng/10^6^ cells) and treated or nontreated with serial dilutions of the test compounds for 48 h. The same concentration of vehicle (DMSO) was used as a nontreated control (100%). The cell culture was then lysed, and RLUs were measured in a luminometer. Results were analyzed using GraphPad software.

The anti-HIV screening was performed by infecting a lymphoblastoid cell line (MT-2) with an X4 tropic recombinant virus (NL4.3-Ren) in the presence of the test compounds at concentrations ranging from 10 to 0.2 μM in base 2 serial dilutions. Mock infected cultures were treated in the same manner to evaluate cytotoxicity. IC_50_ and CC_50_ were calculated for each compound (Table 3).^34^ Compounds **7** and **13** exhibited the most potent activities, with IC_50_ values of 1.9 and 1.0 μM, respectively, similar to the reference drug, tenofovir (IC_50_ 1.7 μM), used as a positive control. Moreover, compounds **1**, **2**, **9**, and **12** displayed moderate activity, with IC_50_ values ranging from 8.9 to 21.1 μM. Regarding their cell toxicity, all the tested compounds were shown to be nontoxic in this model (CC_50_ > 100 μM) (Table 3). The most active compounds, **7** and **13**, exhibited a favorable selectivity index (SI: ratio CC_50_/IC_50_) of 10.6 and 15.5, respectively. Therefore, these two compounds are promising withasteroids and were selected for further elucidation of the mechanism involved in their anti-HIV activity (Table 4, Figure 2).

**Table 3:** Anti-HIV Activity^a^ and Cytotoxicity^b^ of Withasteroids **1**–**13**

compound	IC_50_^a^ (CI95%; *R*^2^^c^) μM	CC_50_^b^ μM	SI^d^
**1**	12.4 (9.5–16.4; 0.8945)	>100	>8
**2**	18.7 (13.6–25.9; 0.8333)	>100	>5.3
**3**	76.1 (44.9–143.6; 0.7311)	>100	>1.3
**4**	72.7 (49.8–111.3; 0.8528)	>100	>1.4
**5**	>100	>100	
**7**	1.9 (0.8–2.7; 0.778)	16.0 (8.4–31.4; 0.8139)	10.6
**8**	67.9 (46.7–103.2; 0.86)	>100	>1.5
**9**	8.9 (6.2–13.1; 0.8256)	>100	>11.2
**10**	32.7 (24.8–43.6; 0.834)	>100	>3.1
**12**	21.1 (14.9–30.1; 0.8075)	>100	>4.7
**13**	1.0 (0.6–1.7; 0.8056)	15.2 (6.8–35.8; 0.7353)	15.5
TFV^e^	1.7 (1.1–2.5; 0.9387)	>100	333

a IC_50_ (inhibitory concentration 50%) values were calculated using GrapPad Pris software. All values are the mean of at least three independent experiments.

b CC_50_ (cytotoxic concentration 50%) values were calculated using GrapPad Pris software. All values are the mean of at least three independent experiments.

c *R*^2^: *R* squared.

d SI: selectivity index (CC_50_/IC_50_).

e TFV: tenofovir used as an antiretroviral drug control.

**Table 4:** Entry Inhibition and Transcriptional Anti-HIV Activity of Withasteroids **7** and **13**

compound	IC_50_ HIV^a^ (CI95%^b^; *R*^c^) μM	IC_50_ VSV-HIV (CI95%; *R*^2^) μM	IC_50_ viral transcription (CI95%; *R*^2^) μM	SI^d^ VSV-HIV/HIV (*p* value)	SI^d^ HIV transcription/HIV infection (*p* value)
**7**	1.94	3.20	1.97	1.64	1.02
	(1.25–2.99; 0.8345)	(1.74–5.88; 0.7624)	(1.38–2.79; 0.8023)	(0.7896)	(0.8079)
**13**	1.01	2.67	1.14	2.66	1.13
	(0.67–1.48; 0.8684)	(1.55–4.56; 0.8136)	(082–1.58; 0,8174)	(0.7408)	(0.6666)
enfuvirtide	0.015	>1	0.01	>66.67	
	(0.009–0.023; 0.9798)		(0.009–0.023; 0.9798)		
TFV	1.69				
	(1.06–2.75; 0.9387)				

a IC_50_ (inhibitory concentration 50%) values were calculated using GraphPad Prism software.

b CI95% (confidence interval 95%).

c *R*^2^: *R* squared.

d Specificity index: SI VSV-HIV/HIV is the relationship between the IC_50_ value obtained in VSV-HIV infection experiments and the IC_50_ from HIV infection experiments. SI HIV transcription/HIV infection is the relationship between the IC_50_ value obtained in transcription experiments and the IC_50_ value from HIV infection experiments. All values are the means of at least three independent experiments. IC_50_ values in HIV infection are repeated here from Table 3 to better compare with values of entry and transcription inhibition.

The influence of the substitution pattern of the withanolide-type skeleton and its relationship with anti-HIV activity (Table 3) were examined, revealing the following trends in this series of natural withanolides: (a) Analysis of the role of functional groups in the B-ring showed that withanolides with an epoxy group at C-5/C-6 (**7**, IC_50_ 1.9 μM) displayed 4.7- and 35.7-fold more potency than those with halohydrin (**9**, IC_50_ 8.9 μM) or dihydroxy (**8**, IC_50_ 67.9 μM) groups. (b) In ring D, a double bond at the C-16 position was highly favorable, since the antiviral effect of **7** was >50-fold more potent than its corresponding 17β-hydroxy-withanolide (**5**, IC_50_ > 100 μM), suggesting that the enhancement of potency could be correlated to the lipophilicity of the molecule. (c) Regarding the lactone ring, the results indicated that substituents at C-24 and C-25 on the core skeleton play a notable role on activity. Thus, compounds **1** and **2** showed better in vitro activity than their congener **5**, suggesting that the hydroxy group at C-24 and the exocyclic double bond at C-25/C-27 may be involved in H-bonding and/or π–π interactions, respectively, with the target. (d) The replacement of the withasteroid typical enone and epoxy systems in the A- and B-rings for the unusual cycloendoperoxide moiety was unfavorable, as withanolides **3** and **4** showed low anti-HIV effects (IC_50_ 76.1 and 72.7 μM, respectively). These trends, based upon substitution patterns, provide valuable information on the pharmacophore for withanolide-type compounds and should be helpful for the rational design of more potent and selective anti-HIV drugs.

In order to investigate a possible mechanism for the anti-HIV effects of the most active compounds, **7** and **13**, a series of additional experiments were performed. First, HIV entry was tested. To do this, VSV envelope pseudotyped HIV (VSV-HIV) was used to infect MT-2 cells in the presence of serial dilutions of these two compounds (base 2 serial dilutions, 100 μM highest concentration) and compared with HIV enveloped virus infections (HIV) (Table 4). Lack of activity against VSV-HIV virus would suggest an HIV envelope dependent activity and, hence, an interference with HIV entry. As shown in Figure 2A and Table 4, both compounds, **7** and **13**, showed IC_50_ values in VSV-HIV infections very close to the values obtained in HIV infections, suggesting a mechanism of action other than HIV entry. In fact, no statistical differences were found between VSV-HIV IC_50_ and HIV IC_50_ values (*p* values higher than 0.7 in all cases, Table 4). On the other hand, enfuvirtide, a CD4-gp120 fusion inhibitor used as control, was not active as a VSV-HIV inhibitor, as expected.

Next, the activities of compounds **7** and **13** on viral transcription (Figure 2B) were analyzed. Viral transcription is a complex process dependent on the cellular transcriptional machinery as well as on viral proteins, such as Tat.

Withasteroids are compounds somewhat related structurally to certain endogenous steroid hormones involved in normal cellular transcription. Since cellular transcription is a key process involved in the development of a successful viral replication cycle, the effect of the compounds on viral transcription was specifically studied. Thus, a luciferase construct under the control of the whole genome of HIV (NL4.3-Luc) was transfected in MT-2 cells and, subsequently, treated with base 2 serial dilutions of these two test compounds, from 0.7 to 100 μM. This model leads to the evaluation of the effect of the test compounds on transcription, overcoming the potential effects produced in previous steps. As shown in Figure 2, withasteroids **7** and **13** inhibited HIV transcription. In fact, transcriptional IC_50_ values were very close to the HIV infection IC_50_ values (SI transcription/infection of 1.02 and 1.13, and *p* values of 8079 and 0.666 for compound **7** and **13**, respectively), suggesting that viral transcription could be the main target of both compounds (Table 4).

Thus, in the current study, and as a continuation of our efforts to find new drug candidates for HIV infection treatment, four new withanolide-type steroids, nicansteroidins A–D (**1**-**4**), and nine known compounds were identified from *P. nicandroides*. Compounds **1** and **2** represent the first naturally occurring examples of withasteroids with a 24-hydroxy-exocyclic-α,β-unsaturated δ-lactone moiety as a distinct structural feature, whereas compounds **3** and **4** are unusual withanolides with a six-membered cyclic peroxide ring system. Biological evaluation revealed that two compounds from this series, **7** and **13**, displayed strong inhibition of HIV-1 replication with IC_50_ values lower than 2 μM. Moreover, cellular mechanism experiments showed that the main target of these compounds in the HIV replication cycle is viral transcription. The present findings provide evidence of **7** and **13** as potential lead compounds for the development of new anti-HIV agents. Their unique architectures and anti-HIV activity provide new insight into the diversity of the withanolide-type steroids and may trigger an increasing interest for further chemical, biosynthetic, and pharmacological investigations.

## Experimental Section

### General Experimental Procedures

Optical rotations were determined on a PerkinElmer 241 automatic polarimeter. UV spectra were obtained in absolute MeOH on a JASCO V-560 spectrometer. IR (film) spectra were measured on a Bruker IFS 55 spectrometer. The 1D and 2D spectra were recorded on a Bruker Avance 600 spectrometer; the chemical shifts are given in δ (ppm) and were referenced to the residual solvent signal (CDCl_3_: δ_H_ 7.26, δ_C_ 77.36; C_6_D_6_: δ_H_ 7.15, δ_C_ 128.62), with TMS as internal reference. EIMS and HREIMS were recorded on a Micromass Autospec spectrometer. Silica gel 60 (15–40 mm) for column chromatography and silica gel 60 F254 for TLC were purchased from Panreac, and Sephadex LH-20 was obtained from Pharmacia Biotech. Centrifugal planar chromatography (CPC) was performed using a Chromatotron instrument model 7924T (Harrison Research Inc., Palo Alto, CA, USA) on manually coated silica gel 60 GF_254_ (Merck) using 1, 2, or 4 mm plates. The developed TLC plates were visualized by UV light and then by spraying with a staining system of H_2_O–H_2_SO_4_–AcOH (1:4:20) followed by heating to approximately 150 °C. All solvents used were analytical grade from Panreac.

### Plant Material

The aerial part of *Physalis nicandroides* Schltdl (Solanaceae) were collected in the Cantón San Nicolás (latitude: 13°54′21″ N, longitude: 88°58′3″ W, elevation: 280 m.a.s.l.), Municipio de Cinquera, Departamento de Cabañas, El Salvador, in July 2017, and the plant was identified by Jenny Elizabeth Menjívar Cruz, curator of the Herbarium at the Museo de Historia Natural de El Salvador. A voucher specimen (Menjívar. J. 4062 Núñez, M. & Hernández R.) was deposited in the Herbarium at the Museo de Historia Natural de El Salvador, El Salvador.

### Extraction and Isolation

The air-dried powdered aerial parts of *P. nicandroides* (384.7 g) were extracted exhaustively with *n*-hexane and then acetone in a Soxhlet apparatus, and the solvent was evaporated at reduced pressure. The acetone extract (21.8 g) was fractionated by liquid chromatography on silica gel and eluted with hexanes–EtOAc mixtures of increasing polarity (from 100:0 to 0:100) and MeOH, affording 22 fractions, which were combined on the basis of their TLC profiles to produce pooled fractions F1 to F8. Preliminary ^1^H NMR analysis revealed fractions F5 to F7 to be rich in withanolides, and these were further investigated. The fractions were subjected repeatedly to column chromatography over Sephadex LH-20 (hexanes–CHCl_3_–MeOH, 2:1:1), silica gel (hexanes–EtOAc of increasing polarity 80% to 100%), CPC, and preparative TLC, using mixtures of hexanes–EtOAc (2:8), hexanes–*i*-propanol (8:2), CH_2_Cl_2_–AcOEt (2:8), or CH_2_Cl_2_–dioxane (7:3), to afford nicansteroidin A (**1**, 3.6 mg), nicansteroidin B (**2**, 5.2 mg), nicansteroidin C (**3**, 6.4 mg), nicansteroidin D (**4**, 3.2 mg), physagulide I (**5**, 17.5 mg), physagulin K (**6**, 0.8 mg), physagulin A (**7**, 23.7 mg), withaminimin (**8**, 9.3 mg), physagulin B (**9**, 12.3 mg), physagulin J (**10**, 10.7 mg), (20*S*,22*R*)-15α-acetoxy-5α-chloro-6β,14β-dihydroxy-1-oxowitha-2,24-dienolide (**11**, 0.7 mg), physagulin C (**12**, 8.0 mg), and physagulin F (**13**, 4.7 mg).

#### Nicansteroidin A (**1**):

colorless lacquer; [α]^20^_D_ +31.0 (*c* 0.48, MeOH); UV λ_max_ (log ε) 215 nm; IR (film) ν_max_ 3435, 2924, 2851, 1717, 1672, 1378, 1247, 756 cm^–1^; ^1^H NMR (CDCl_3_, 400 MHz), see Table 1; ^13^C NMR (CDCl_3_, 400 MHz), see Table 2; EIMS *m*/*z* 544 [M^+^] (1), 526 (1), 466 (8), 448 (22), 420 (11), 315 (21), 251 (29), 124 (100), 109 (63); HREIMS *m*/*z* 544.2681 [M^+^] (calcd for C_30_H_40_O_9_, 544.2672).

#### Nicansteroidin B (**2**):

colorless lacquer; [α]^20^_D_ +23.0 (*c* 0.47, MeOH); UV λ_max_ (log ε) 212 nm; IR ν_max_ 3431, 2926, 2854, 1716, 1671, 1458, 1398, 1247, 756 cm^–1^; ^1^H NMR (CDCl_3_, 400 MHz), see Table 1; ^13^C NMR (CDCl_3_, 400 MHz), see Table 2; EIMS *m*/*z* 544 [M^+^] (1), 526 (1), 466 (10), 448 (29), 430 (12), 315 (29), 251 (37), 236 (50), 135 (90), 124 (100); HREIMS *m*/*z* 544.2666 (calcd for C_30_H_40_O_9_, 544.2672).

#### Nicansteroidin C (**3**):

colorless lacquer; [α]^20^_D_ +1.6 (*c* 1.48, MeOH); UV λ_max_ (log ε) 213 nm; IR ν_max_ 3480, 2924, 2852, 1734, 1712, 1376, 1224, 755 cm^–1^; ^1^H NMR (CDCl_3_, 400 MHz), see Table 1; ^13^C NMR (CDCl_3_, 400 MHz), see Table 2; ESIMS (positive) *m*/*z* 565 [M + Na]^+^ (100); HRESIMS *m*/*z* 565.2415 [M + Na]^+^ (calcd for C_30_H_38_O_9_Na, 565.2414).

#### Nicansteroidin D (**4**):

colorless lacquer; [α]^20^_D_ +0.7 (*c* 1.0, MeOH); UV λ_max_ (log ε) 214 nm; IR ν_max_ 3435, 2928, 1722, 1680, 1378, 1237, 757 cm^–1^; ^1^H NMR (CDCl_3_, 400 MHz), see Table 1; ^13^C NMR (CDCl_3_, 400 MHz), see Table 2; EIMS *m*/*z* 558 [M^+^] (1.4), 524 (3), 480 (4), 428 (9), 341 (11), 267 (25), 225 (85), 125 (99), 55 (1000); HREIMS *m*/*z* 558.2474 (calcd for C_30_H_40_O_10_, 558.2465).

#### Physagulide I (**5**):

colorless lacquer; [α]^20^_D_ +8.3 (*c* 0.31, MeOH); UV λ_max_ (log ε) 212 nm; ^1^H NMR (CDCl_3_, 400 MHz), see Table 1; ^13^C NMR (CDCl_3_, 400 MHz), see Table 2; EIMS *m*/*z* 544 [M^+^] (1), 526 (1), 466 (8), 448 (22), 430 (11), 315 (21), 251 (29), 124 (100), 109 (63); HREIMS *m*/*z* 544.2666 (calcd for C_30_H_40_O_9_, 544.2672).

### Biological Assays

MT-2 cells were cultured in RPMI 1640 medium containing 10% (v/v) fetal bovine serum, 2 mM l-glutamine, penicillin (50 IU/mL), and streptomycin (50 μg/mL) (all Whittaker Bio-Products, Walkerville, MD, USA). 293T cells were cultured in DMEM medium containing 10% (v/v) fetal bovine serum, 2 mM l-glutamine, penicillin (50 IU/mL), and streptomycin (50 μg/mL) (all Whittaker Bio-Products). MT-2 and 293T cells were cultured at 37 °C in a 5% CO_2_ humidified atmosphere and split twice a week.

#### Plasmids and Virus

Plasmids pNL4.3-Ren and pNL4.3-Luc were generated by cloning the renilla or luciferase genes in the nef site of pNL4.3.^34^ VSV-HIV supernatants were obtained by cotransfection of pNL4.3-Luc-R^–^E^–^, a full length HIV DNA that does not express the HIV envelope (obtained from NIH AIDS Research and Reference Reagent Program, NIAID, NIH) and pcDNA-VSV, DNA for vesicular stomatitis virus (VSV) G glycoprotein cloned in the pcDNA3.1 plasmid.^35^

#### Anti-HIV Evaluation

The anti-HIV activity of compounds was evaluated by a recombinant virus assay (RVA) and performed as follows: infectious supernatants were obtained from calcium phosphate transfection on 293T cells of plasmids pNL4.3-Ren or cotransfection of the pNL4.3-Luc-R^–^E^–^, full-length HIV-DNA that does not express the HIV envelope, and pcDNA3-VSV, which expresses the G-protein of VSV. These supernatants were used to infect MT-2 cells (100 000 RLUs/well or 20 ng of Gag-p24 protein per well) in the presence or absence of different concentrations (base 2 serial dilutions) of the test compounds being evaluated. After 48 h in culture at 37 °C and 5% CO_2_, cell cultures were lysed with 100 μL of buffer with “reporter lysis buffer” (Promega, Madison, WI, USA). Relative luminescence units (RLUs) were obtained in a luminometer (Berthold Detection Systems, Pforzheim, Germany) after the addition of substrate provided by the luciferase assay system kit (Promega) to cell extracts. Viability was evaluated in mock-treated cells with the same conditions of the RVA. Cell viability was measured using the CellTiter Glo assay system (Promega), following the manufacturer’s specifications. Inhibitory concentrations 50 (IC_50_) and cytotoxic concentrations 50% (CC_50_) were calculated using GraphPad Prism v9.0 software.

#### Viral Entry Evaluation Assay

To evaluate viral entry, MT-2 infections performed with recombinant wild type HIV (NL4.3-Ren) (100 000 RLUs per well or 20 ng of p24 per well) were compared with a VSV-pseudotyped recombinant HIV (HIV-VSV) (100 000 RLUs per well or 20 ng of p24 per well), which enter the target cells through a CD4/CXCR4 or CCR5 receptor independent mechanism. The methodology is similar to the antiviral activity evaluation. Infections were performed with both viruses (HIV and VSV-HIV) in parallel, and, after 48 h, RLUs were obtained in a luminometer. Enfuvirtide, a CD4-gp120 fusion inhibitor, was used as a reference control of viral entry inhibition. IC_50_ was calculated using GraphPad Prism v9.0 (GraphPad software).

#### Transfection Assays

MT-2 cells were maintained in culture without stimuli and collected prior to the assay in RPMI without serum and antibiotics. MT-2 cells were then suspended in 350 μL of RPMI without supplements and subjected to electroporation using an Easyject Plus electroporator (Equibio, Harrow, UK) at 260 V, 1500 mF, and maximum resistance with 1 μg/106 cells of a luciferase plasmid under the control of the whole genome of HIV-1 (pNL4.3-Luc). Afterward, MT-2 cells were seeded in 24-well microplates and treated with different concentrations of compounds, base 2 serial dilutions, using phorbol myristate acetate (PMA) as a reference control of HIV-1 transactivation, and left in culture in complete RPMI at 37 °C. Then, 48 h later, cultures were lysed with luciferase buffer (Luciferase assay system buffer, Promega) and luciferase activity (RLUs) was measured in a luminometer (Berthold Detection Systems).

#### Statistical Analysis

The statistical analysis was performed using GraphPad Prism v9.0 software. Data were treated using Microsoft Excel software, and IC_50_ and CC_50_ values calculated using a nonlinear regression analysis and dose–response inhibitions curves formula; *t* test analysis was performed to calculate the significant differences (*p* value) between HIV IC_50_ values, both using GraphPad Prism v9.0 (GraphPad software).

## Supporting Information Available

NMR spectra for natural withasteroids **1**–**5** (PDF)
