# Supplementary material for: Withanolide-Type Steroids from Physalis nicandroides Inhibit HIV Transcription
Source: J Nat Prod. 2021 Sep 22;84(10):2717–26. doi: 10.1021/acs.jnatprod.1c00637 (PMC8946265; doi:10.1021/acs.jnatprod.1c00637)
Supplement: Supplementary file 1 — np1c00637_si_001.pdf [file np1c00637_si_001.pdf]

Electronic Supporting Information

Withanolide-type steroids from *Physalis nicandroides* inhibit HIV transciption

Vito A. Taddeo,[†,‡] *Marvin J. Núñez,*[§] *Manuela Beltrán,*[O] *Ulises G. Castillo,*[§] *Jenny Menjívar,*[^] *Ignacio A. Jiménez,*[†] *José Alcamí,*[O] *Luis M. Bedoya,*[O,#,*] *Isabel L. Bazzocchi*[†,*]

[†] Instituto Universitario de Bio-Orgánica Antonio González and Departamento de Química Orgánica, Universidad de La Laguna, Avenida Astrofísico Francisco Sánchez 2, 38206 La Laguna, Tenerife, Spain.

[‡] Dipartimento di Farmacia, Università degli Studi "G. d'Annunzio" Chieti-Pescara, Via dei Vestini 31, 66100 Chieti, Italy.

[§] Laboratorio de Investigación en Productos Naturales, Facultad de Química y Farmacia, Universidad de El Salvador, Final Av. de Mártires y Héroes del 30 de Julio, San Salvador 1101, El Salvador.

[O] Retrovirus Laboratory, Department of AIDS Immunopathogenesis, National Centre of Microbiology, Instituto de Salud Carlos III, Ctra. Pozuelo Km. 2. 28220, Majadahonda, Madrid, Spain.

[^] Museo de Historia Natural de El Salvador, Ministerio de Cultura, San Salvador 1101, El Salvador.

[#] Pharmacology, Pharmacognosy and Botany Department, Pharmacy Faculty, Universidad Complutense de Madrid, Pz. Ramón y Cajal s/n, 28040, Madrid, Spain.

Table of contents

S1. ^{1}H NMR spectrum (600 MHz, $CDCl_3$) of compound **1**

S2. ^{13}C NMR spectrum (150 MHz, $CDCl_3$) of compound **1**

S3. HSQC spectrum (600 MHz, $CDCl_3$) of compound **1**

S4. HMBC spectrum (600 MHz, $CDCl_3$) of compound **1**

S5. ROESY spectrum (600 MHz, $CDCl_3$) of compound **1**

S6. ^{1}H NMR spectrum (600 MHz, $CDCl_3$) of compound **2**

S7. ^{13}C NMR spectrum (150 MHz, $CDCl_3$) of compound **2**

S8. ^{1}H NMR spectrum (600 MHz, $CDCl_3$) of compound **3**

S9. ^{13}C NMR spectrum (150 MHz, $CDCl_3$) of compound **3**

S10. HSQC spectrum (600 MHz, $CDCl_3$) of compound **3**

S11. HMBC spectrum (600 MHz, $CDCl_3$) of compound **3**

S12. ROESY experiment (600 MHz, $CDCl_3$) of compound **3**

S13. ^{1}H NMR spectrum (600 MHz, $CDCl_3$) of compound **4**

S14. ^{13}C NMR spectrum (150 MHz, $CDCl_3$) of compound **4**

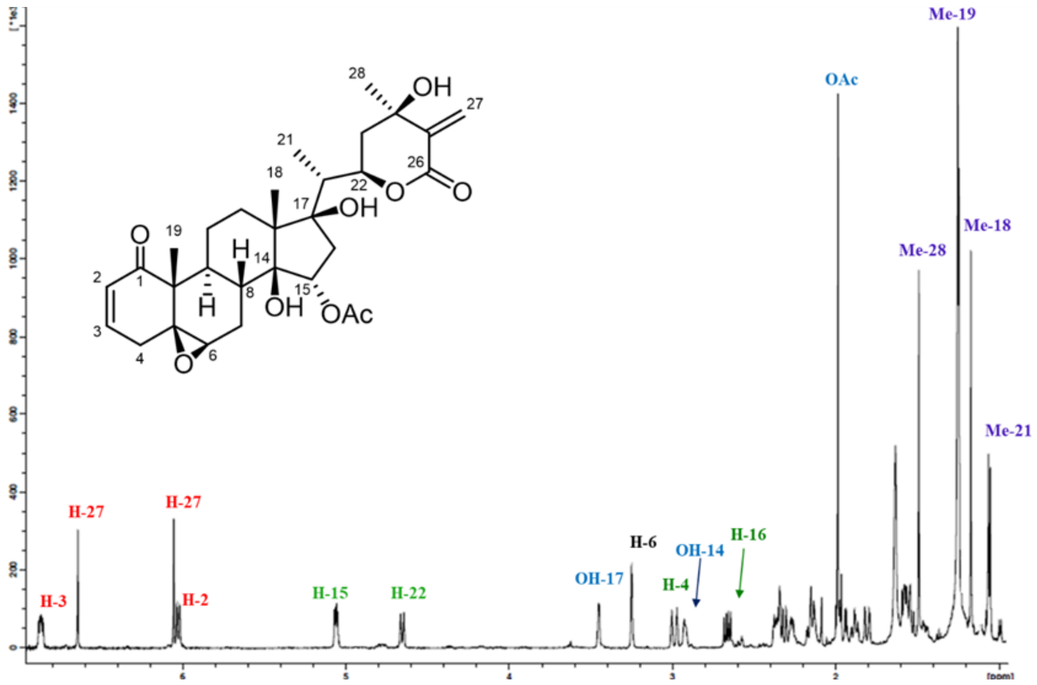

S1. ^{1}H NMR spectrum (600 MHz, $CDCl_3$) of compound **1**

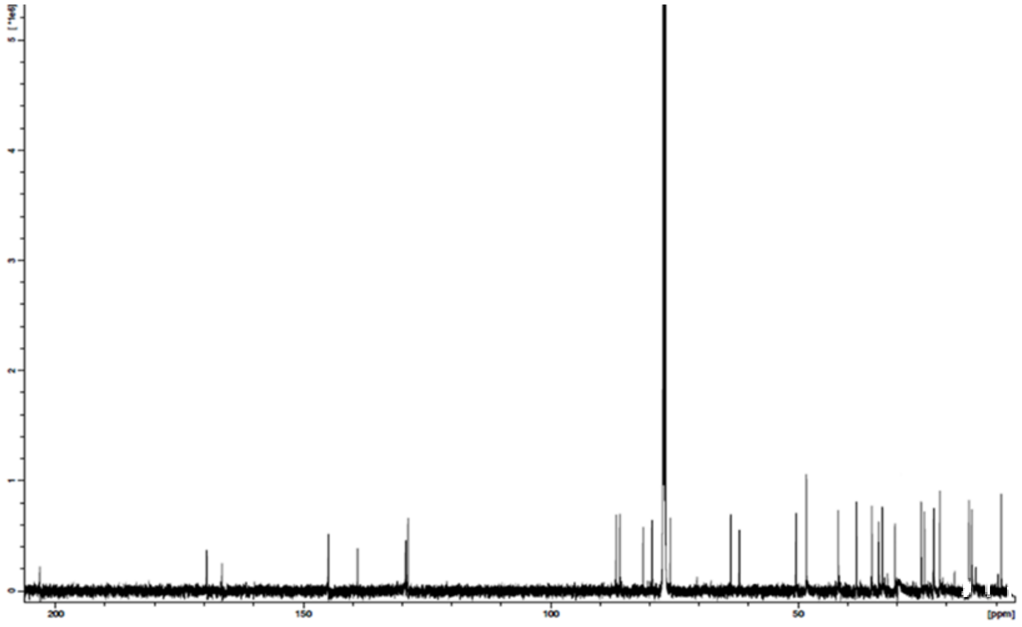

S2. ^{13}C NMR spectrum (150 MHz, $CDCl_3$) of compound **1**

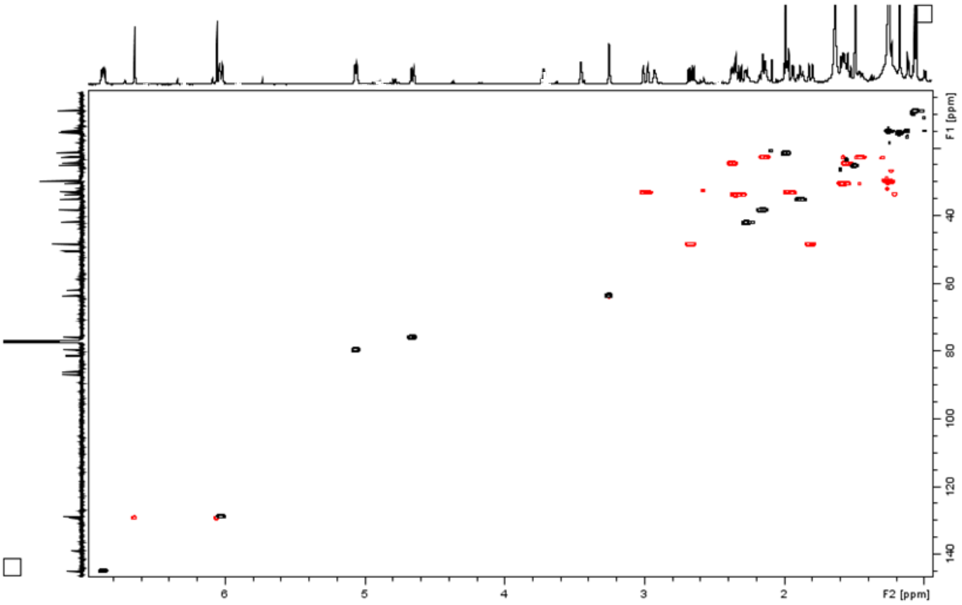

S3. HSQC spectrum (600 MHz, $CDCl_3$) of compound **1**

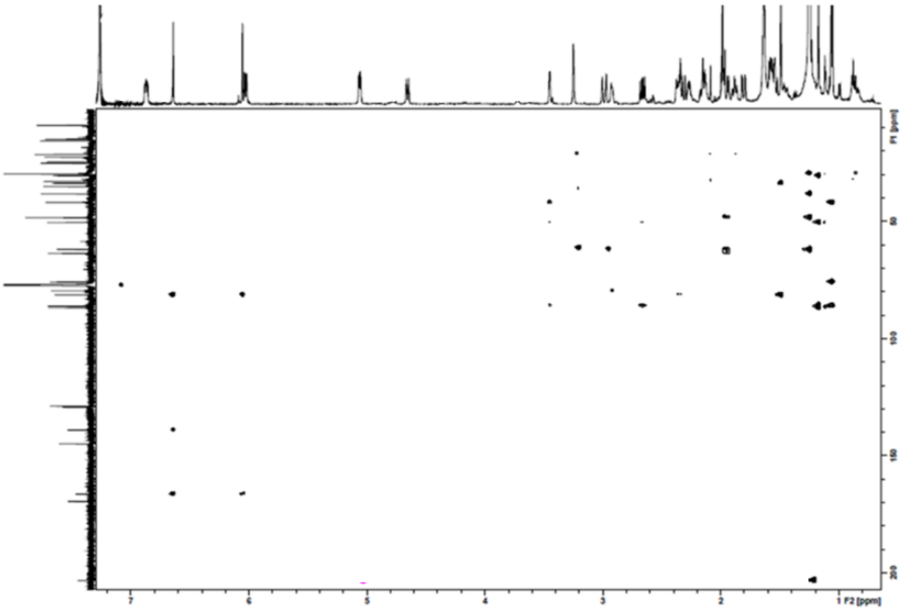

S4. HMBC spectrum (600 MHz, $CDCl_{3,}$) of compound **1**

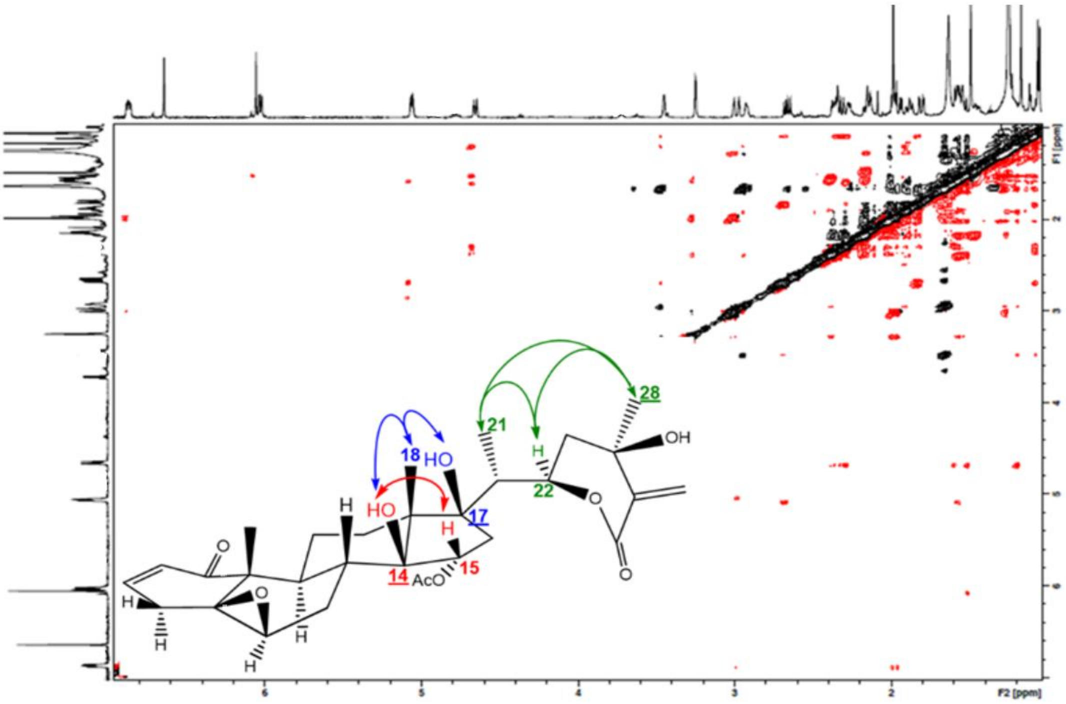

S5. ROESY experiment (600 MHz, $CDCl_3$,) of compound **1**

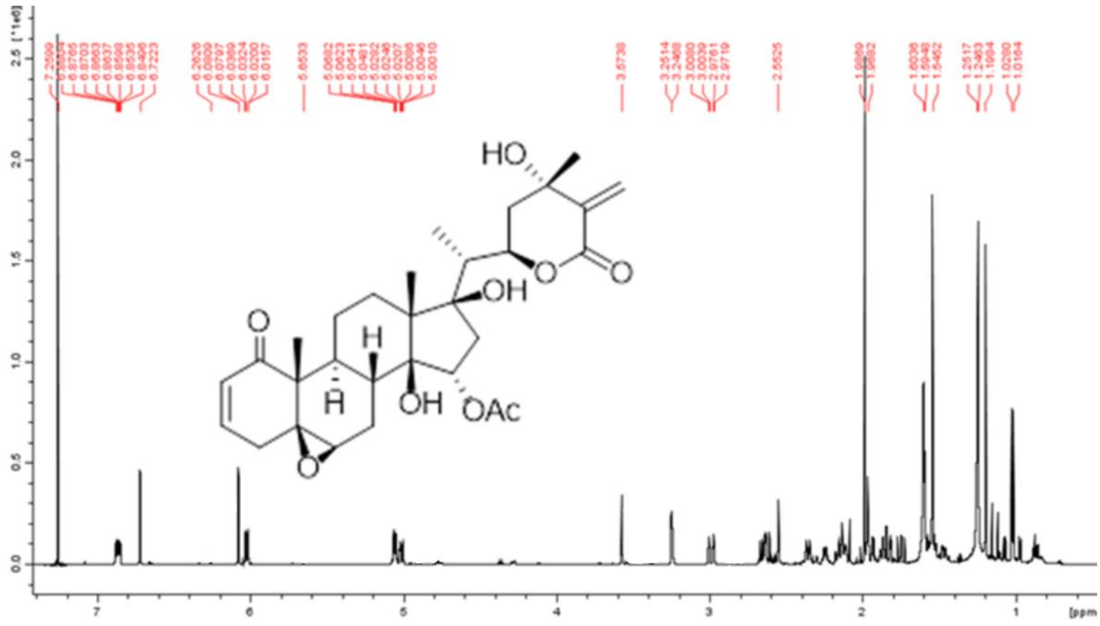

S6. ^{1}H NMR spectrum (600 MHz, $CDCl_3$) of compound **2**

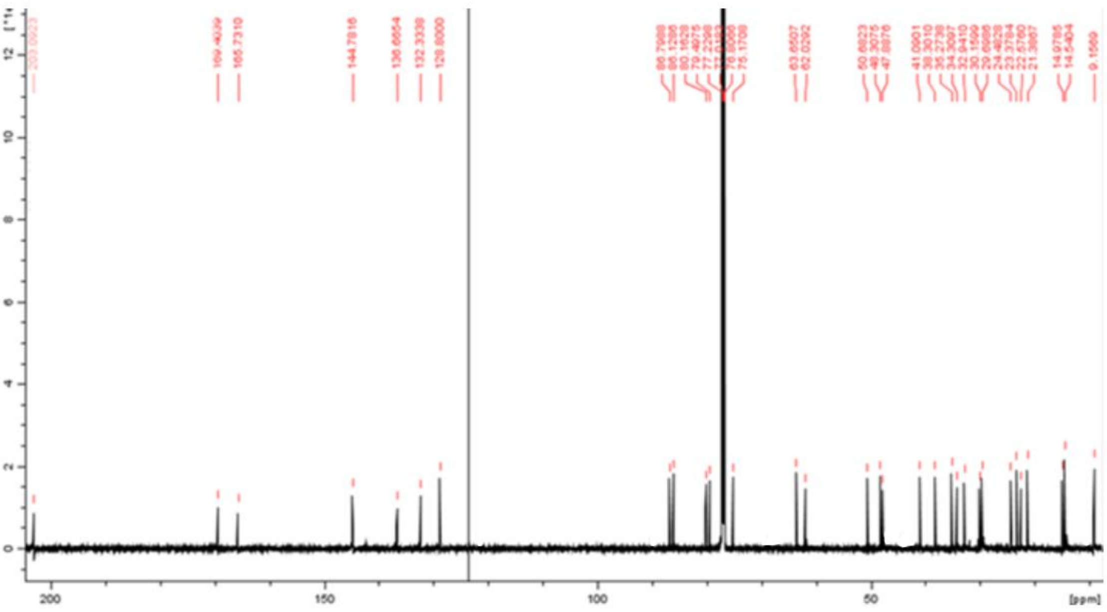

S7. ^{13}C NMR spectrum (150 MHz, $CDCl_3$) of compound **2**

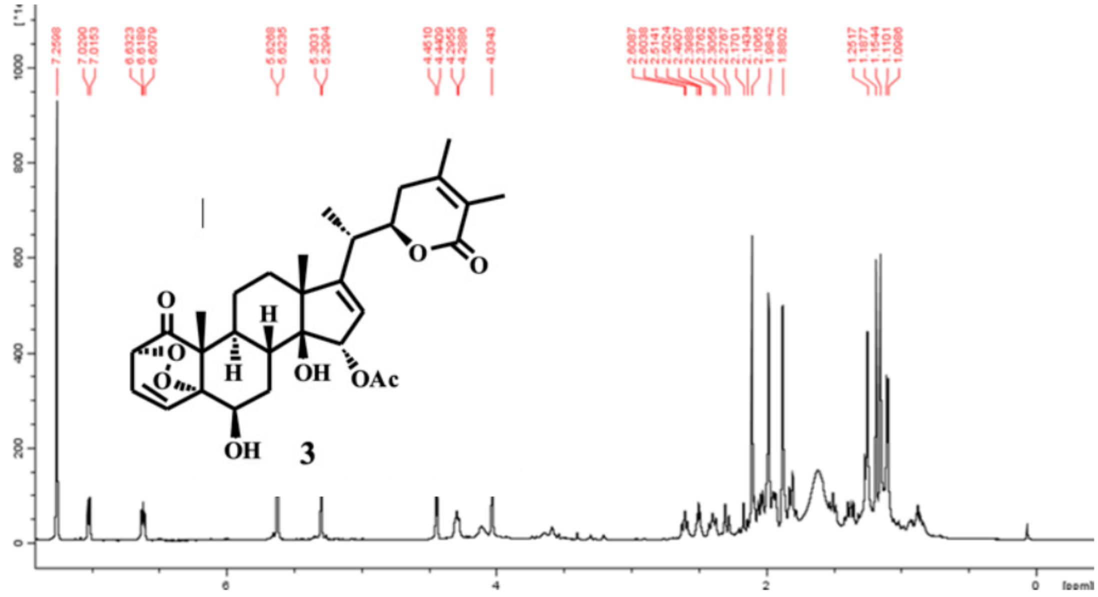

S8. ^{1}H NMR spectrum (600 MHz, $CDCl_3$) of compound **3**

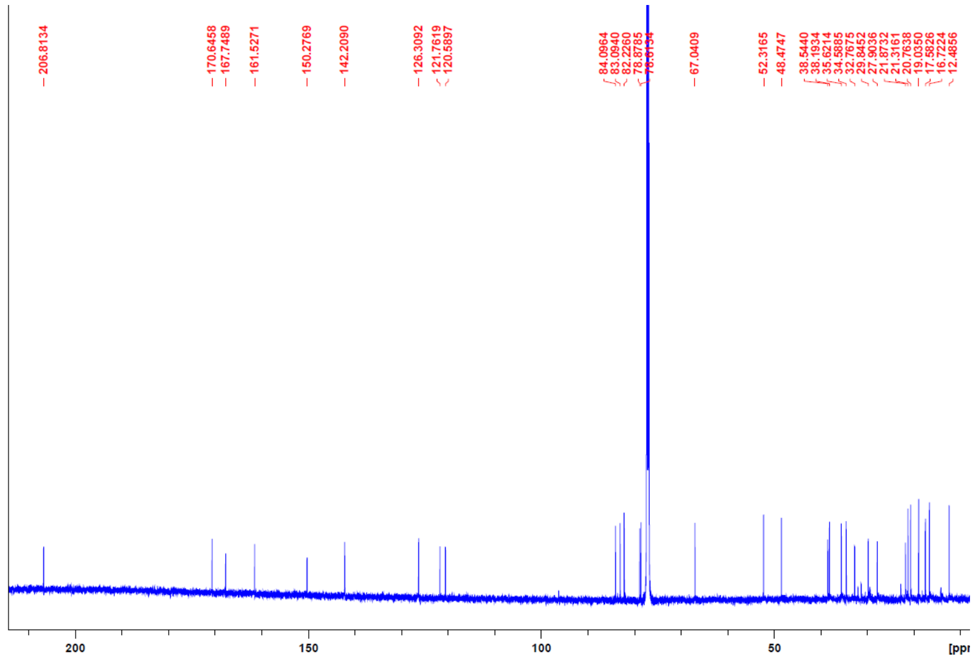

S9. ^{13}C NMR spectrum (150 MHz, $CDCl_3$) of compound **3**

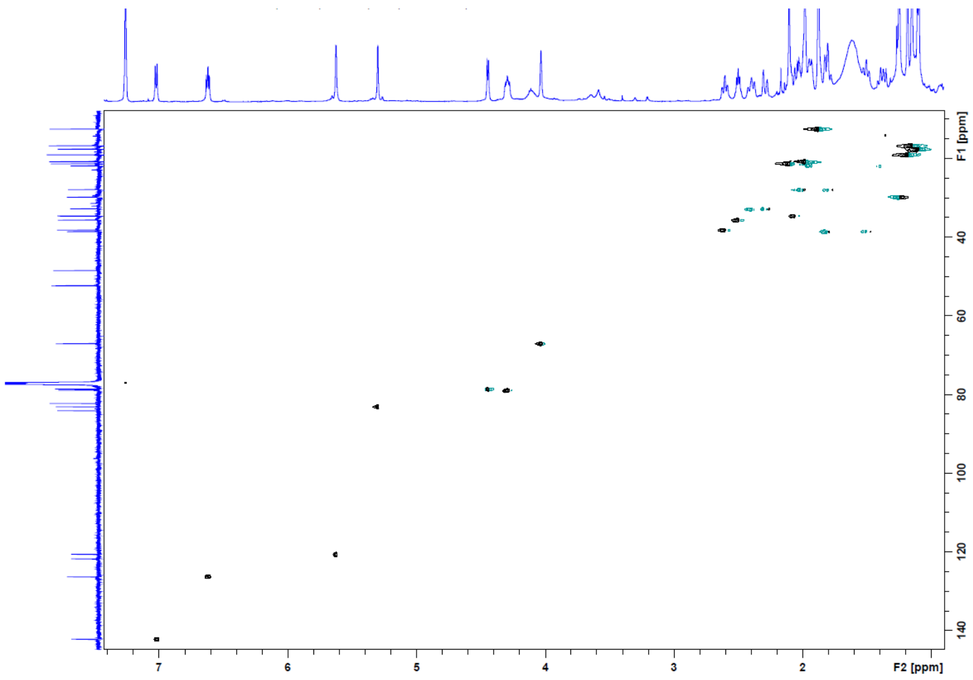

S10. HSQC spectrum (600 MHz, $CDCl_3$) of compound **3**

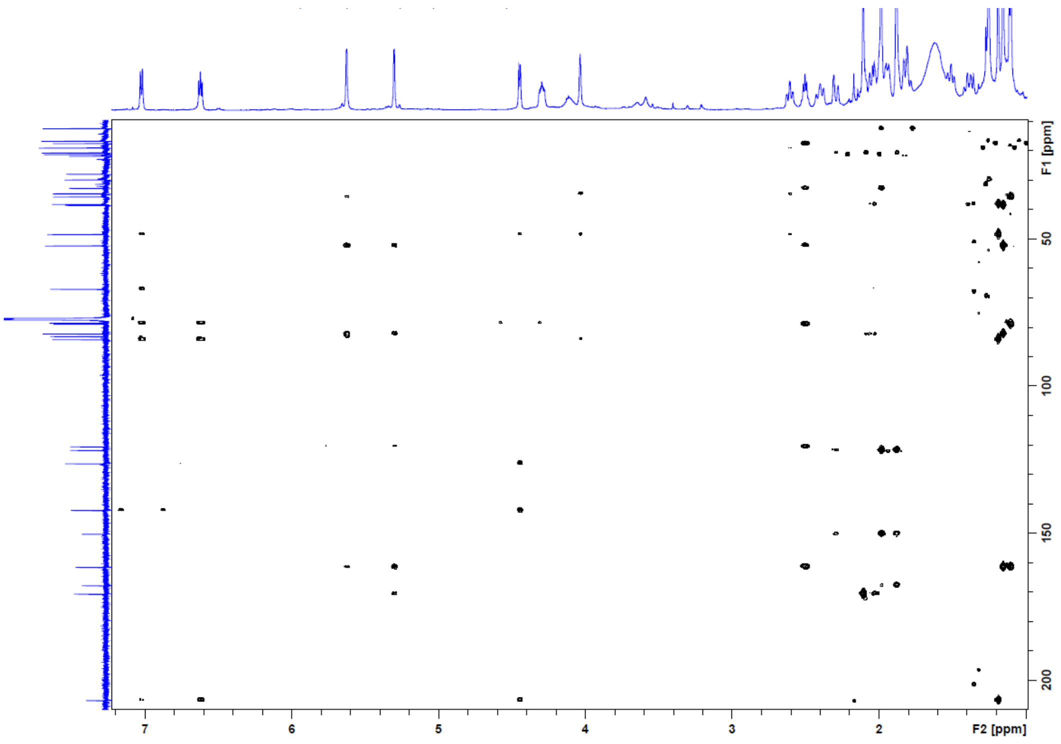

S11. HMBC spectrum (600 MHz, $CDCl_3$) of compound **3**

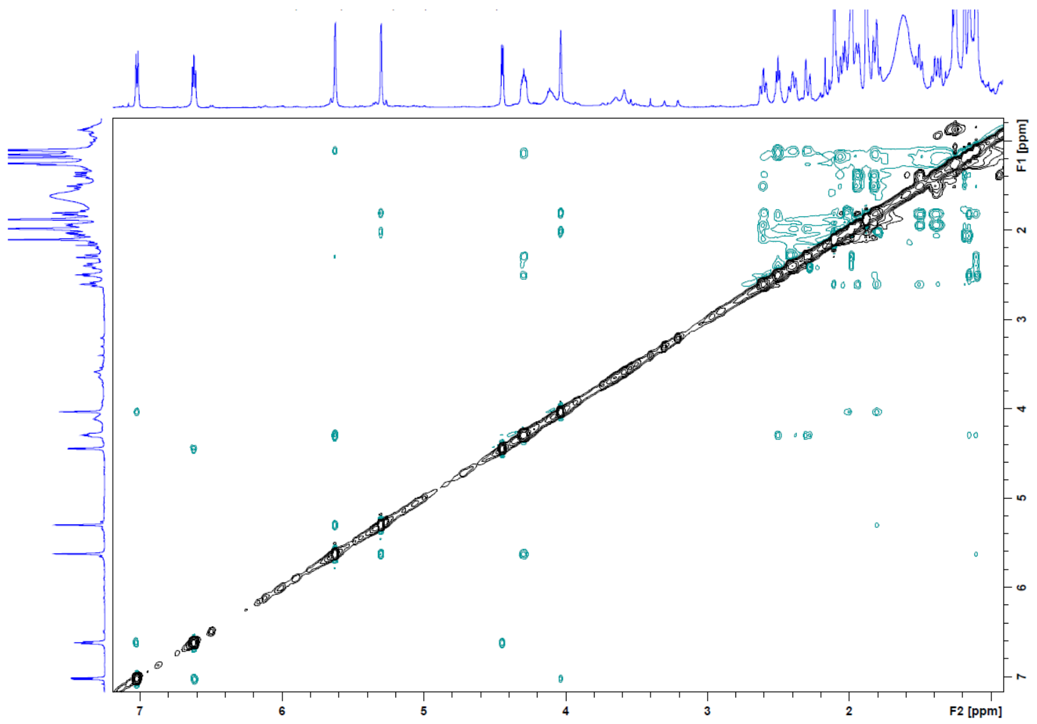

S12. ROESY experiment (600 MHz, $CDCl_3$,) of compound **3**

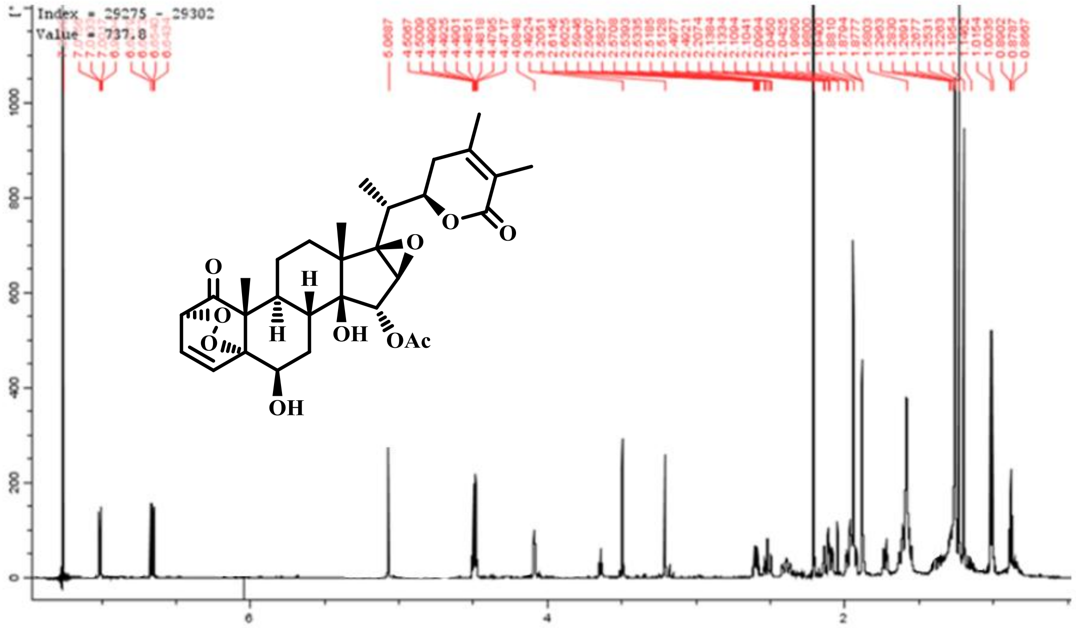

S13. ^{1}H NMR spectrum (600 MHz, $CDCl_3$) of compound **4**

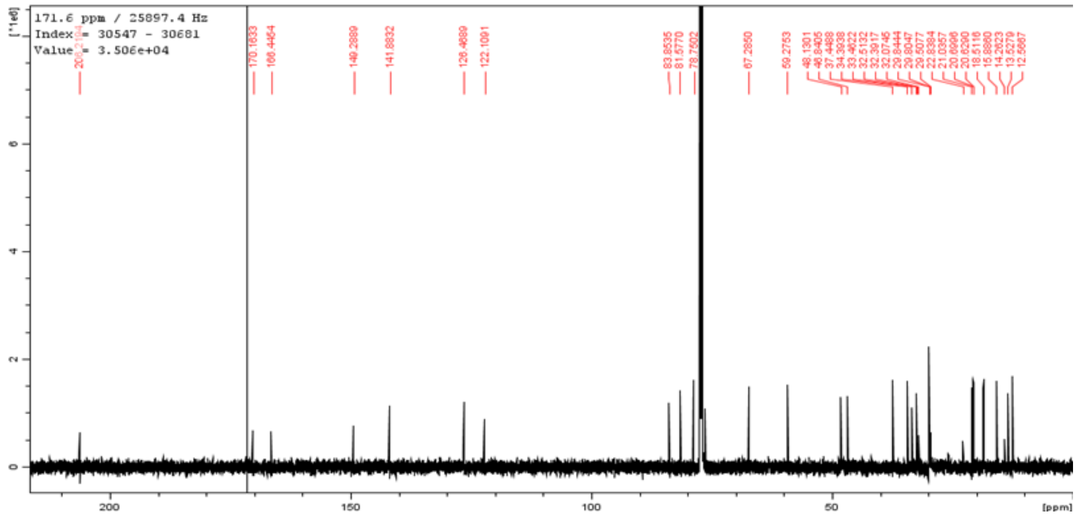

S14. ^{13}C NMR spectrum (150 MHz, $CDCl_3$) of compound **4**
